# Involution and governance reform in professional football club–school integration: a case study of S City’s youth training base

**DOI:** 10.3389/fphys.2025.1557875

**Published:** 2025-04-30

**Authors:** Lin Wang, Chuanfang Zhu, Yuyuan Zhang, Lan Wang

**Affiliations:** ^1^ School of Physical Education, Huangshan University, Huangshan, China; ^2^ School of Foreign Languages, Huangshan University, Huangshan, China; ^3^ School of Financial Government, Zhejiang Financial College, Hangzhou, China

**Keywords:** professional football club, campus football, football reserve talents, integration of sports and education, youth football training base

## Abstract

The integrated development of Chinese professional football clubs and campus football has achieved good development. However, there are still many problems in terms of training methods and depth of integration. In this article, we use the involution theory (a developmental paradox where increased partnership networks fail to improve training outcomes) as an analytical perspective to explain the inner part of the case. We identify an involution dilemma in football club–school integration, characterized by overemphasis on quantitative expansion (increasing partner schools across provinces) and underinvestment in training quality (neglecting youth base development). The findings reveal a systemic “growth without development” pattern, where scaling partnerships fails to enhance talent outputs. The specific causes of the involution dilemma are stated in detail in this manuscript. Based on this, the research proposes specific corrective strategies to solve these issues.

## 1 Introduction

The integration of professional football clubs and school football programs has emerged as a critical strategy for sustainable talent development in global football. This paradigm shift responds to the growing recognition that traditional youth development systems often fail to balance athletic excellence with educational attainment and personal development (Yu et al., 2025). The fusion of professional sports expertise with educational institutions’ structured learning environments offers a holistic approach to nurturing well-rounded athletes. Globally, this integration movement has gained momentum, with various nations developing unique models tailored to their sociocultural contexts. In Japan, the J-League’s “School-Club Linkage Program” has established over 200 partnerships, significantly improving youth player retention rates (Xiang and Xu, 2008). The United States’ “Development Academy” system, although recently restructured, demonstrated the potential of combining elite training with academic flexibility (Shu and Yu, 2015). It has become an important consensus in deepening the integration and development of professional football clubs and campus football to cultivate competitive football talent. This not only optimizes the practical foundation for the development of campus football but also contributes to expanding the pool of potential talent for the youth training teams of professional football clubs (Yu and Liu, 2019; Yu et al., 2019; Qiu and Qin, 2021). In recent years, China’s integrated development of professional football clubs and school football has achieved significant progress, with youth training bases serving as a crucial linkage mechanism. As of June 2022, China had designated 32,780 school football model schools, demonstrating initial success in grassroots promotion. A four-tier league system (primary to university levels) and a parallel summer camp structure were established, engaging over 20 million student participants by December 2022, with 60,000+ student-athletes annually attending regional and national camps. Further systemic development includes the “Starry Sky” Elite Training Program, linking schools with youth academies. By June 2023, 15 women’s football academies were operational nationwide, each partnering with ≥20 local schools as talent pipelines. However, comprehensively integrating the development of professional football clubs with that of campus football to train competing football talents is still in an early exploratory stage, with most issues regarding training methods or how to deepen integration.

Taking S City’s S Professional Football Club, for example, in recent years, the S Professional Football Club has widely expanded its cooperation in campus football to establish a youth football training base. However, the integrated development exhibits a pronounced involution trend, characterized by an overemphasis on quantitative expansion at the expense of qualitative improvement. Specifically, the current approach prioritizes scaling up partnership networks while insufficiently investing in enhancing existing youth training infrastructure. This disproportionate focus has created a significant disparity between the scope of collaboration and its substantive outcomes. This thesis takes the Youth Football Training Base of the S Professional Football Club in S City as a case to analyze the restrictive factors in the process of integration and development so as to further deepen and improve the effectiveness of school–campus integration.

Based on the theory of involution, this thesis analyzes its manifestation in this case, explains the logic behind the formation of the involution dilemma, and proposes corresponding optimization paths. It also offers theoretical guidance for the integration and development of other domestic professional football clubs and campus football.

## 2 Theoretical basis

### 2.1 Analysis of the applicability of involution theory and research

#### 2.1.1 Overview of involution theory

It should be noted that the formation and emergence of involution theory underwent a prolonged historical process (Jiangfeng, 2019). The current research generally attributes the origin of the concept of involution to Immanuel Kant. In his attempt to distinguish “evolution” from “progression,” involution first appeared in the Critique of Judgment completed by Kant. Later, the theory was incorporated into anthropology and agricultural economics. It gained prominence with the publication of Clifford Geertz’s Agricultural Involution: The Processes of Ecological Change in Indonesia in 1963. This book not only popularized the concept of involution in academic circles but also established its significance in agricultural economics. Geertz argued that administrative obstacles in Java prevented agricultural development from shifting to a capital-intensive model, as seen on other islands, thus confining it to a labor-intensive mode and resulting in agricultural involution on Java Island (Clifford, 1963). Since then, involution theory has found widespread application in political science, economics, and sociology. For example, it was argued in Huang Zongzhi’s concept of “economic involution” that in late Qing rural China, the increase in family income was due to the intensive use of family labor rather than an increase in daily wages per worker. This growth, stagnant and inward-focused, became a significant obstacle to the transformation of the smallholder economy into industrial development in the late Qing society. American scholar Du Zancheng proposed the idea of “political involution,” suggesting that the early Chinese Republic was marked by the national government replicating and extending existing institutions without enhancing the efficiency of either old or new state institutions. In such cases, it can be seen as a demonstration of “state power involution.” As the applicability of involution theory has expanded, scholars have provided increasingly accessible interpretations of its meaning. The core idea of involution is “growth without development” (Ji and Yuanxin, 2020), representing an undesirable form of transformation and evolution (He and Cai, 2005); it is a process of development without actual progress or benefit, characterized by the continuous repetition and replication of old mechanisms (Guo, 2021).

Recent years have witnessed increasing application of involution theory in education and grassroots governance research. Studies suggest that involution may become a persistent feature of grassroots governance, with its manifestations varying across historical periods due to spatiotemporal constraints (Li, 2024). Future governance research must address the long-term coexistence of involution and de-involution dynamics, seeking pathways to overcome involutionary traps and achieve developmental governance.

In education, involution characterizes as phenomena of excessive competition, epitomizing contemporary educational pathologies. It reveals dual dilemmas, namely, externally meaningless expansion and internally self-consuming development, while reflecting the constrained existential conditions of educational participants (Li and Wang, 2025). Fundamentally, involution stems from finite educational resources as a prerequisite, with irrational competition exacerbating its severity—ultimately leading to human self-alienation.

In the sports domain, involution manifests distinctive characteristics. Under multifactorial influences, sports organizations establish relatively rigid developmental models with stringent regulatory mechanisms, leading to path dependence and self-locking effects during organizational evolution. Consequently, organizational development degenerates into prolonged incremental progress or even stagnation (He and Liu, 2022). Taking Chinese martial arts as a case study, external constraints have driven increasing internal complexity and refinement without facilitating substantive progress. Instead, this traditional sport demonstrates developmental stagnation, failing to achieve genuine innovation while undergoing gradual decrease, weakening, and cultural displacement (Li and Wang, 2018).

#### 2.1.2 Analysis of research applicability

Based on the review of relevant research on involution, it is clear that the core essence of involution is “growth without development.” The development path lacks substantial gains, and the development stage does not progress to a higher level. From the perspective of case development practice, integrating and developing the S Professional Football Club with campus football in practice coincides with the core essence of involution and demonstrates a marked tendency toward involution.

In the process of integration and development, although efforts have been made to increase collaboration with other provinces and cities, insufficient investment has been directed toward cultivating youth football training bases. When considering the path to development, the plan has consistently focused on expanding campus football collaboration with other cities, rather than pursuing deeper and more effective integration. This approach has resulted in a failure of deep integration. The proportion of skilled football players produced through campus football programs remains low, preventing them from becoming a reliable and steady source for the youth training team at the S Professional Football Club to select talented players. Consequently, the connection between professional football clubs and university football remains in a state of “growth without development.”

### 2.2 PSR model and the reasons of involution

The PSR model, standing for “Pressure-State-Response,” was first introduced as the “Stress-Response” framework by Canadian statisticians Tony Friend and David Rapport in 1979, which was used to analyze the interaction between the natural environment and the human social system (Rapport and Friend, 1979). In this context, “pressure” refers to human production and lifestyle activities, whereas “response” refers to the natural and socioeconomic reactions to these activities. In 1991, the Organisation for Economic Co-operation and Development (OECD) and the United Nations Environment Programme (UNEP) refined and developed the “Pressure-Response” model for environmental quality assessment (Author anonymous, 2003). They subsequently divided the “response” element into “state” and “response,” creating the “Pressure-State-Response” model and establishing a theoretical framework for sustainable development based on the logic of “causes-effects-measures.” In environmental quality assessment, “pressure” refers to the impacts of human activities on natural resources and ecosystems, including the various changes and conditions of environmental elements caused by human actions, whereas “response” refers to the policies and measures implemented by governments, businesses, NGOs, and the public. The clear causal relationships and logical progression of the PSR model are effective in illustrating dynamic processes influenced by multiple factors, helping answer the questions “Why?,” “What?,” and “What to do?” (Xie and Huang, 2017). Over time, the PSR model has been applied across various fields, including politics, economics, society, culture, ecology, and sports, with the specific contents of “pressure,” “state,” and “response” evolving to reflect the characteristics of each field.

It logically allows for the development of the root causes of involution and paths of mitigation by providing its “causes-effects-measures” logical chain. The PSR model’s cause-effect-measure logic better captures systemic pressures in transitional sports systems than CIPP’s program evaluation focus. Based on the previous observation, the given thesis tries to develop certain reasons for involution within a presented case by referring to a more problem-oriented PSR analytical framework, on whose foundation the path should be considered that will enable escaping the involution dilemma.

## 3 Methodology

In this study, we use in-depth interviews (n = 21) with key stakeholders to explore the integration process, analyzed through qualitative coding methods. The interviewees specifically include the following: one youth training director from the S Club, one youth training coach from the club, two primary school physical education teachers from two primary schools in S city, two primary school principals, and 15 physical education teachers and principals from primary schools outside S city. The interview with the youth training coaches from the S Professional Football Club is organized to ascertain information about the club’s goals, development status, and strategies adopted toward establishing youth football training bases. The interviews with the local and external campus football teachers are designed to collect information on the integration model and the specific support measures of their schools in cooperation with the S Professional Football Club.

A qualitative research approach was employed in this study, with semi-structured interviews for data collection. However, no specialized qualitative data analysis software (e.g., NVivo or MAXQDA) was used for data processing. To ensure data integrity and accuracy, the research worker obtained informed consent from all participants and recorded the entire interview process using professional audio equipment. Following the interviews, the audio recordings were meticulously transcribed verbatim, and the textual data were cross-checked repeatedly to ensure transcription accuracy. A traditional content analysis method was applied, involving manual coding to systematically categorize, organize, and analyze the textual data.

## 4 Case overview and involution dilemma

### 4.1 Case basic information

S City’s S Professional Football Club is a relatively young club in East China. It has consistently ranked in the upper-middle tier of the Chinese Football Association Super League (CSL) and has previously won the CSL championship. Since 2015, the club has developed its youth training program through partnerships with schools and football training bases across China. By leveraging the large number of students involved in campus football training, the club identifies top talent to strengthen its youth team.

#### 4.1.1 Case integration and development model

The S Professional Football Club adopts a “decentralized to centralized” training and management model for its nationwide network of youth football training bases. “Decentralized” refers to the training management of student players at their respective schools. During the school term, after-school training is conducted by school football teachers, with training content consisting of teaching videos and syllabi specially designed by the club’s youth training department. The “centralized” aspect refers to the gathering of all student players from the nationwide youth football training bases at the club’s own facility during winter and summer breaks for intensive training. During these breaks, the club also invites players from its youth football training bases to participate in national and international youth football events.

In the “centralized” model, organizing youth football events is a key training method. The S Professional Football Club collaborates with various stakeholders to organize a series of regular youth football events. These events serve two purposes: providing competition opportunities for players from the national youth football training bases and evaluating the training levels at these bases while identifying outstanding campus football talent.

Among these events, the summer camp of the nationwide youth football training base, organized by the S Professional Football Club and its parent company, is the largest and most comprehensive training activity.

The summer camp includes youth football competitions as the main activity, with supporting activities such as organizing visits for teachers and students to watch important matches of the first team, including those in the Chinese Super League, AFC Champions League, and FA Cup. Additionally, there are meet-and-greet sessions with star players and football coaching workshops. In the management and development of cooperative school football teachers, apart from arranging special training sessions for youth coaches in the summer camp, it also arranges regular annual youth coaching seminars every year to enhance the teaching capabilities of football instructors.

#### 4.1.2 Specific measures and resource support for case integration and development

While managing the youth football training bases, the S Professional Football Club provides a series of resource supports to the partner schools. In terms of resource types, these include the following: football technical guidance for teachers and students; donations for football equipment, such as jerseys, footballs, and training tools; opportunities for competitive events; funding for football development; and role model motivational support. Role model motivational support refers to the visits by the S Professional Football Club’s star players to schools for interaction with teachers and students. These role models can use their status to motivate the student players to try harder and train harder. However, for the different partner schools, the types and frequency of support from the S Professional Football Club are also different. In another word, local partner schools gain relatively more resources than the partner schools out of the province or city.

### 4.2 Reality representation of the involution dilemma in the case

The involution dilemma herein lies in balancing two strategic approaches: expanding inter-provincial campus football partnerships versus deepening existing collaborative effectiveness.

This involution dilemma manifests as an overemphasis on expanding cross-provincial partnerships at the expense of youth academy development—a strategic imbalance that successful models like Bundesliga youth academies have demonstrably avoided (Güllich, 2013). Specifically, German professional clubs establish football schools within campuses to deliver specialized training (Güllich, 2013) while maintaining a structured age-group system (U9–U19) to optimize talent development (Naglo, 2020). Institutionally, the German Football Association (DFB) actively facilitates club–school collaborations through policy formulation, resource allocation, and coach education programs (Hornig et al., 2016).

In contrast, China’s current approach prioritizes quantitative expansion of training infrastructure over qualitative refinement of developmental systems. Since 2015, when the S Professional Football Club initiated its nationwide youth football training base program, the club has been making significant investments in expanding the scope of these training bases, establishing over 50 across the country. However, until now, no campus football players have been promoted to professional football clubs. Compared with the success rate of the German youth training system, the effect is not obvious. Among the established youth football training bases, most are in partnership with schools in other provinces and cities, with only three bases directly established in collaboration with local schools. Although most youth football training bases of the S Professional Football Club are distributed in schools from other provinces and cities, this club has displayed obvious “differentiated treatment” in terms of investment in training of students at these respective training bases. This is specifically reflected in two aspects: support for resource elements and the management approach to training.

First, in terms of resource support: for local youth football training bases, the S Professional Football Club provides comprehensive resource support, including full-process football technical guidance for both teachers and students, donations for football equipment, development of funding, motivational support from role models, and opportunities for competitive events. For youth football training bases in other provinces and cities, resource support is mainly provided through competitive event opportunities. Although there is some technical guidance for teachers and students, it is limited to “concentrated” training events. In contrast, local youth football training bases receive continuous technical guidance during both the “dispersed” and “concentrated” training phases. Additionally, when local schools prepare for major national or regional campus football competitions, the S Professional Football Club sends youth training coaches for intensive coaching. Although football equipment donations and role model motivational support are typically provided at the start of the partnership, the frequency of support is generally higher for local schools.

Second, concerning the method of training management: the S Professional Football Club also adopts different management approaches in training students at local versus out-of-province youth football training bases. In the case of local youth football training bases, the S Professional Football Club sends professional youth training coaches to regularly conduct on-site teaching and training. Training guidance for youth football in out-of-province schools, however, is managed through a school-based model, with implementation by local school football teachers. On the whole, campus football out-of-province development and training depend on providing opportunities for competitive events. Although event opportunities are indeed one of the important resources currently lacking in the development of campus football and are an important factor affecting the improvement of students’ football skills, from the point of view of training football talent, teaching consistent practice, and frequent competition are all essential components in developing high-level athletes (Wang, 2020; Liu et al., 2021; Mao et al., 2021).

### 4.3 Specific causes of the involution dilemma

**FIGURE 1:**
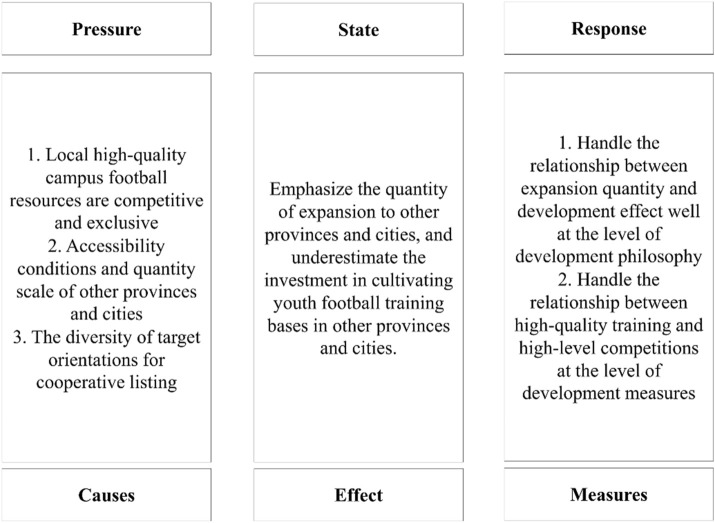
PSR model of the S Professional Football Club collaborating with campus football to establish a youth football training base.

This strategic misalignment manifests in two core issues: 1. why does it focus on expanding the number of youth football training bases in other provinces and cities? 2. What are the reasons that have led the S Professional Football Club to neglect the investment in student players at these out-of-province youth football training bases? The thesis uses the PSR model developed by the European Environmental Agency, an important analysis framework with the natural world in the perspective of external pressure, states, and response, while establishing a PSR model to the S Professional Football Club’s cooperation with campus football to establish youth football training bases (Figure 1). This model is to explain the causes of the involution dilemma and its correction strategies. The “State” in this model is the real-world representation of the involution dilemma analyzed above, which is to emphasize the expansion of quantity in other provinces and cities while ignoring investment in training out-of-province youth football training bases. “Pressure” refers to the specific causes leading to the involution dilemma, whereas “response” refers to the strategies adopted in an attempt to eliminate and correct the involution dilemma.

First, the competitiveness and exclusivity of high-quality local campus football resources are the primary reasons for the expansion of youth football training bases to other provinces and cities. There are two professional football clubs in S City, neither of which has its own affiliated youth training school. The long-term challenge of selecting talented players for their youth teams has hindered the development of the youth training systems at both clubs. To ease the difficulty of player selection, both clubs have been exploring cooperation in campus football to establish youth football training bases for developing reserve talent, particularly since the implementation of the “Professional Football Club Admission Regulations (2018 Edition)” by the Chinese Football Association, which introduced the “team bundling club registration system,” and the subsequent requirement for CSL and CLO clubs to establish at least five youth teams (U19, U17, U15, U14, and U13), whereas CLT clubs must establish at least four youth teams (U17, U15, U14, and U13) [15]; the collaboration between the two professional football clubs and campus football has accelerated.

The plan to develop a youth football training base offers a new solution for selecting talent for the youth teams of two professional clubs; however, local high-quality campus football resources are limited. From an economic perspective, campus football cooperation partners are competitive and exclusive, indicating a single school cannot partner with both professional clubs to jointly establish a youth football training base. This is because the other club initiated its youth football training base plan earlier, and most of the local high-quality campus football cooperation partners had already established relationships with that club.

The S Professional Football Club is relatively late in developing its youth football training base and has established partnerships with only three local schools so far. Consequently, the club has extended its youth football training bases to other provinces and cities.

Moreover, the accessibility and scale of these bases in other regions have impacted the S Professional Football Club’s investment in player development. The training bases set up through campus football cooperation are primarily located in schools outside S City. These bases are numerous but relatively inaccessible. The scale and accessibility directly influence how the S Professional Football Club manages the “distributed” training at these external bases. As a result, the club can only provide remote, school-based management, meaning that regular, on-site teaching is not feasible. The concept of school-based management involves indirect instruction. In practice, the S Professional Football Club creates special teaching videos and training syllabi for nonlocal bases, which are provided to local coaches for training student players. Ideally, these materials should positively impact the players’ football development. However, it is widely recognized that the coaching ability of grassroots campus football teachers is generally low (Wang and Fan, 2018). As a result, after teachers learn and internalize the materials, they may struggle to train the students effectively, potentially leading to uncontrollable training quality and poor learning outcomes. Consequently, the effectiveness of player development is significantly reduced by the school-based management model alone, resulting in a very low success rate for student players at external bases. Campus football cooperation aims to identify talent for youth teams. However, if the external school-based training bases cannot reliably and consistently serve as a source of talent, the S Professional Football Club, from an economic “rational actor” perspective, is unlikely to invest in these external bases. In contrast, the three local campus football partners in S City offer good accessibility and allow the club to provide regular, hands-on coaching directly in the schools. These three schools have long been established as football powerhouses in the region, producing professional and national team players. They have also achieved significant success in national and regional campus football competitions in recent years. Therefore, in terms of accessibility and foundational development, local schools are more suitable partners for the S Professional Football Club’s campus football development. Following the principle of “rationality” and maximizing mutual benefits, the S Professional Football Club naturally prioritizes its resources on local campus football.

## 5 Implications for research

Based on the analysis of the “involution” dilemma in this case, two corrective strategies are proposed in this thesis, using the PSR model as the analytical framework.

First, the expansion of collaborations should be balanced with the achievement of development outcomes. Development concepts guide actions, and all practices are led by specific ideas. The appropriateness of the development concept fundamentally determines the success or failure of the practice. In terms of integrating development concepts, the primary focus should be on cultivating high-quality football talent for competitive sports.

Considering the issues highlighted in the case, it is crucial to balance the quantity and effectiveness of campus football cooperation. In the practice of the S Professional Football Club and other professional clubs integrating with campus football, “land-grabbing” for campus football resources is emerging. Although the quantity of campus football cooperation is essential for integrated development, it should not result in meaningless growth. The foundation of integrated development should address the problems and gaps in campus football that schools cannot manage independently, providing detailed professional training to student players to improve their competitive football skills, thereby developing suitable candidates for youth team selection.

The establishment of youth football training bases should not focus solely on increasing the number of collaborations or football players. It should not be about “signing partnerships without fostering development.” The S Professional Football Club should adopt a pragmatic, focused development plan, dedicating more effort to improving the football skills of student players. A coordinated approach is necessary, ensuring that the increase in partnerships aligns with tangible player development outcomes.

Based on comprehensive analysis, the club should now prioritize a “quality-first” strategic transition, focusing on in-depth cultivation within existing partner schools to establish a refined talent development system.

Second, the high-level training should be related to the high-level competition in development projects. Teaching, consistent practice, and frequent competition are all indispensable means to enhance the competitive football skills of campus football players (China edu net, 2021). The S Professional Football Club organized a series of regular youth football events during “concentrated” training periods, providing ample competitive opportunities for national youth football training bases. However, significant issues remain in the teaching and training at youth football bases in other provinces and cities. Although competitive opportunities are essential for improving the football skills of student players, daily high-quality training is the fundamental requirement for cultivating competitive football talent. This is a key shortcoming in other regions. Therefore, for the youth football training measures in other provinces and cities, a balance between teaching/training and competition must be struck. While continuing to organize various youth football events, the club should also enhance the level and international competitiveness of these competitions to offer more challenging opportunities for players at all training bases. Additionally, the club should increase the frequency of in-school training services at youth football bases in other provinces, encouraging professional players to visit schools during their free time to provide guidance. For schools in regions with limited accessibility where the club cannot offer regular in-school training, both parties should explore innovative methods for delivering training. The club could co-finance and partner with local youth training organizations, supported by the community, to provide training services.

## 6 Study applicability and limitations

### 6.1 Theoretical framework and applicability

In this study, we develop an innovative analytical framework based on involution theory to systematically examine the integration efficiency between professional football clubs and campus youth training systems in China. The framework provides novel insights into the “growth without development” dilemma in sports governance.

The findings primarily apply to resource-constrained small- and medium-sized professional clubs, particularly those without self-established youth academies. Furthermore, the framework offers valuable implications for other emerging football markets undergoing rapid expansion (annual growth rate >15%), serving as a reference for avoiding developmental inefficiencies.

### 6.2 Limitations

This qualitative case study of the Professional Football Club in City S, primarily using interview and observational data, presents two key methodological constraints: first, the single-case research design may compromise the external validity of findings, whereas the absence of quantitative data impacts measurement precision. Second, the study period coincided with ongoing structural reforms in Chinese football (e.g., updated youth academy regulations), potentially affecting the temporal generalizability of conclusions. We recommend future investigations adopt mixed-methods approaches incorporating quantitative analysis and multi-case comparisons to enhance the robustness and generalizability of findings. (Data collection period: 2020–2023; informants included three stakeholder groups: club executives, coaches, and academy players.).

## 7 Conclusion

The integration of professional football clubs with campus football effectively bridges sports and education systems. This synergy enhances school football participation, creates athlete career pathways, and cultivates youth players to professional academies annually. However, in actual development, some professional football clubs are restricted by objective factors such as the external competitive environment, the finiteness of resources, and the geographical reach of their support capabilities. Moreover, subjective factors like the values and goal orientations of the clubs also contribute to the distortion from the ideal top-level design, leading to a distorted development pattern. Therefore, to promote a better integration between professional football clubs and campus football, analyzing the internal and external situation of each professional football club and their cooperating schools in depth is quite necessary, thus forming targeted, operable, and personalized solutions.

## Data Availability

The raw data supporting the conclusions of this article will be made available by the authors, without undue reservation.
